# Human Polyomavirus JC monitoring and noncoding control region analysis in dynamic cohorts of individuals affected by immune-mediated diseases under treatment with biologics: an observational study

**DOI:** 10.1186/1743-422X-10-298

**Published:** 2013-09-30

**Authors:** Anna Bellizzi, Elena Anzivino, Donatella Maria Rodio, Sara Cioccolo, Rossana Scrivo, Manuela Morreale, Simona Pontecorvo, Federica Ferrari, Giovanni Di Nardo, Lucia Nencioni, Silvia Carluccio, Guido Valesini, Ada Francia, Salvatore Cucchiara, Anna Teresa Palamara, Valeria Pietropaolo

**Affiliations:** 1 Department of Public Health and Infectious Diseases, Sapienza University, P.le Aldo Moro, 5, 00185 Rome, Italy; 2 Department of Internal Medicine and Medical Disciplines, Rheumatology, Sapienza University of Rome, Rome, Italy; 3 Department of Medico-Surgical Sciences and Biotechnologies, Section of Neurology, Sapienza University of Rome, Rome, Italy; 4 Department of Neurology and Psychiatry, Sapienza University of Rome, Rome, Italy; 5 Department of Pediatrics, Pediatric Gastroenterology and Liver Unit, Sapienza University of Rome, Rome, Italy; 6 Department of Public Health and Infectious Diseases, Institute Pasteur, Cenci-Bolognetti Foundation, Sapienza University of Rome, Rome, Italy; 7 Department of Biomedical, Surgery and Dental Sciences, University of Milan, via Pascal 36, 20123 Milan, Italy; 8 San Raffaele Pisana Scientific Institute for Research, Hospitalization and Health Care, Rome, Italy; 9 Sbarro Institute for Cancer Research and Molecular Medicine, Center for Biotechnology, College of Science and Technology, Temple University, Philadelphia, Pennsylvania, USA

**Keywords:** JCPyV, q-PCR, JCPyV-specific antibodies, Multiple sclerosis, Natalizumab, Chronic inflammatory rheumatic diseases, Crohn’s disease, anti-TNF, NCCR, VP1

## Abstract

**Background:**

Progressive multifocal leukoencephalopathy (PML) onset, caused by Polyomavirus JC (JCPyV) in patients affected by immune-mediated diseases during biological treatment, raised concerns about the safety profile of these agents. Therefore, the aims of this study were the JCPyV reactivation monitoring and the noncoding control region (NCCR) and viral protein 1 (VP1) analysis in patients affected by different immune-mediated diseases and treated with biologics.

**Methods:**

We performed JCPyV-specific quantitative PCR of biological samples collected at moment of recruitment (t0) and every 4 months (t1, t2, t3, t4). Subsequently, rearrangements’ analysis of NCCR and VP1 was carried out. Data were analyzed using χ^2^ test.

**Results:**

Results showed that at t0 patients with chronic inflammatory rheumatic diseases presented a JCPyV load in the urine significantly higher (p≤0.05) than in patients with multiple sclerosis (MS) and Crohn’s disease (CD). It can also be observed a significant association between JC viruria and JCPyV antibodies after 1 year of natalizumab (p=0.04) in MS patients. Finally, NCCR analysis showed the presence of an archetype-like sequence in all urine samples, whereas a rearranged NCCR Type IR was found in colon-rectal biopsies collected from 2 CD patients after 16 months of infliximab. Furthermore, sequences isolated from peripheral blood mononuclear cells (PBMCs) of 2 MS patients with JCPyV antibody at t0 and t3, showed a NCCR Type IIR with a duplication of a 98 bp unit and a 66 bp insert, resulting in a boxB deletion and 37 T to G transversion into the Spi-B binding site. In all patients, a prevalence of genotypes 1A and 1B, the predominant JCPyV genotypes in Europe, was observed.

**Conclusions:**

It has been important to understand whether the specific inflammatory scenario in different immune-mediated diseases could affect JCPyV reactivation from latency, in particular from kidneys. Moreover, for a more accurate PML risk stratification, testing JC viruria seems to be useful to identify patients who harbor JCPyV but with an undetectable JCPyV-specific humoral immune response. In these patients, it may also be important to study the JCPyV NCCR rearrangement: in particular, Spi-B expression in PBMCs could play a crucial role in JCPyV replication and NCCR rearrangement.

## Background

The introduction of biologics in the management of immune-mediated disease, such as multiple sclerosis (MS), chronic inflammatory rheumatic diseases (CIRDs) and Crohn’s disease (CD), has been recently associated with the development of serious side effects, such as the progressive multifocal leukoencephalopathy (PML) onset, caused by human Polyomavirus JC (JCPyV) reactivation from oligodendrocytes [1]. JCPyV is a neurotropic double-stranded DNA virus isolated in 1971 from the brain of a patient with Hodgkin’s disease [2]. The 50–90% of adult human population seem to be seroconverted to the virus, with 19–27% of these people shedding JCPyV in their urine [3,4]. The JCPyV genome is composed of early and late regions that are physically separated by the non-coding control region (NCCR). The early region encodes for proteins (TAg, tAg and T primes) involved in viral gene regulation and replication, whereas the late genes encode for the viral capsid proteins (VP1, VP2 and VP3) [5]. The coding regions are well conserved and they are associated with various viral subtypes, which can be found in different geographical areas [6]. Conversely, the NCCR sequence is hyper-variable and contains neurotropic and neurovirulent determinants. The rearranged NCCR sequences, forming during immunosuppression, correlate with poor clinical outcome in PML patients. PML is a demyelinating disease of the brain, originally recognized as a rare complication of hematological malignancies or systemic inflammatory disorders. However, a dramatic 50-fold increase in the PML incidence occurred with the HIV epidemic [5,7]. Recently, cases of PML were also observed in patients under treatment with biologics, including monoclonal antibodies (mAbs). In particular, the mAb natalizumab (Tysabri®; Biogen Idec, Elan Pharmaceuticals) that targets the very late antigen-4 (VLA-4), a cell adhesion molecule, to prevent extravasation of inflammatory T cells into tissues, have been associated with PML [8]. Furthermore, several cases of demyelinating events of the nervous system have been reported for anti-tumor necrosis factor (TNF) agents, such as infliximab (Remicade®; Centocor Ortho Biotech), in CD, severe forms of plaque psoriasis, rheumatoid arthritis (RA) and spondyloarthritis [9-11]. Considering the significant impact of PML onset on the development of biologic agents, it is imperative to assess the risk of PML associated with these therapies in order to achieve a well-informed risk/benefit decision-making. Recently, it was calculated that the risk of PML was of 11.1 cases per 1000 patients (95% CI, 8.3 to 14.5) among natalizumab-treated individuals who had all three main risk factors: presence of anti-JCPyV antibodies, prior use of immunosuppressants, and natalizumab treatment for 25 to 48 months [12-14]. It was also hypothesized that natalizumab could block lymphocyte trafficking through the blood-brain barrier, resulting in a decrease of immunosurveillance that allows JC virus reactivation from latency [15]. Moreover, possibly as a consequence of a blocking effect of this drug on the VLA4/vascular cell adhesion molecule 1 (VCAM-1) interaction, a rapid egress of CD34+ cells from the bone marrow (BM) was observed [16-18]. To date, there are no methods that can reliably predict which patients have a higher risk of developing PML, and no clear-cut associations can be established between JCPyV DNA in the blood or urine and PML [18-22]. In the attempt to bypass this problem, a new assay (STRATIFY JCV®) was suggested to be a sensitive tool to identify JCPyV seroconverted patients [23]. Complementary tests of JCPyV DNA in urine were also indicated as being useful for the stratification of risk of PML in patients [24], since the detection of urinary viral DNA identifies JCPyV infected subject when antibody are still undetectable [25].

Conversely, among patients with CIRDs, 34 confirmed and biological agents-related cases of PML have been reported on the Food and Drug Administration Adverse Event Reporting System (FDA AERS) database, and 17 of them had a diagnosis of systemic lupus erythematosus (SLE). These results are consistent with previous observations [26-29] that patients with SLE appear to have a particular susceptibility to the development of PML, respect to other patients with CIRDs. Moreover, six reports described PML occurrence in CIRDs patients treated with anti-TNF therapies, although all these cases were confounded by treatment with other immunosuppressive agents. Consequently, the rarity of the association between anti-TNF therapy and PML represent an evidence against a causal role of anti-TNF therapy in the development of PML. However, the recent report of an apparently non-confounded case of PML in an RA patient treated with infliximab calls for caution [30].

To date, the precise mechanism by which these treatments may have facilitated the pathogenesis of PML is a matter of debate, but likely it is based on leukocytes inability to traffic to the sites of JCPyV reactivation and infection, and on trafficking of JCPyV latently infected B cells and/or haematopoietic precursors CD34+ between the BM and the brain. JCPyV activity in sites of persistence, and ultimately development of PML, is restricted by viral NCCR variation and activation of gene expression from the NCCR by essential cellular factors [31]. Since JCPyV latency is associated with cells undergoing haematopoietic development, it is probable that transcription factors reported as lymphoid specific regulate JCPyV gene expression. One such factor, Spi-B, is up-regulated in peripheral blood mononuclear cells (PBMCs) in response to treatment with natalizumab. Spi-B is also involved in differentiation and maturation of B cells and it is expressed at high levels in developing and mature B cells. Recently, Spi-B has been reported to bind the NCCR of the PML-associated Mad-1 and Mad-4 but not the non-pathogenic archetype CY, in specific binding sites adjacent to TATA boxes. The active Spi-B-binding site of Mad-1/Mad-4 differs from that of CY by a single nucleotide mutation, strongly affecting the early viral gene expression [32]. Importantly, similar mutation that creates active Spi-B-binding sites adjacent to the TATA box of archetype-like JCPyV have been described by our research group in intestinal biopsies of CD patients treated with infliximab, suggesting that this mutation is supported during dissemination in the host [33].

Therefore, in order to understand the JCPyV reactivation trend in patients with immune-mediated diseases treated or candidates to treatment with biologics, we have enrolled three cohorts: a cohort of MS patients (Cohort1) treated with natalizumab, a cohort of CIRDs patients (Cohort2) treated with conventional immunosuppressants and candidates to biologics, and a cohort of CD patients (Cohort3) treated with infliximab. The first aims of this study have been the JCPyV detection by quantitative Real Time PCR (q-PCR) in several samples collected from the three cohorts at specific sampling times. Subsequently, we have performed the analysis of the possible rearrangements of JCPyV NCCR, in order to detect cellular transcription factors binding site mutations, and the study of JCPyV VP1 sequence, in order to define a possible correlation between a specific JCPyV genotype/subtype and immune-mediated diseases and/or biological treatments.

## Results

### Clinical outcome

A clearly positive outcome of the study was that no PML cases developed during the follow-up period in any cohort.

Regarding the Cohort1, clinical relapses during the 12-month period of therapy were observed in only 1/21 MS patients. However, only eighteen patients reached a follow-up of 12 months, because one patient suspended natalizumab for allergic reaction and two patients showed poor compliance. Moreover, one patient suspended the biological therapy for uterine cancer and papillomatosis after 11 months of natalizumab infusions, and another one did not perform two natalizumab infusions because of *Escherichia coli* urinary infection (Table 1). The clinical efficacy of natalizumab was confirmed by the observation that the Kurtzke Expanded Disability Status Scale (EDSS) [34] remained stable in all 18/21 patients (86%).

**Table 1:** JCPyV-DNA q-PCR in plasma, urine and PBMCs samples of 21 MS patients in 1-year follow-up

		Plasma*^,^**				Urine*^,^**				PBMCs*^,^**				
**Pts.**	**STRATIFY JCV® t0†**	**t0**	**t1**	**t2**	**t3**	**t0**	**t1**	**t2**	**t3**	**t0**	**t1**	**t2**	**t3**	**STRATIFY JCV® t3†**
MS 1	**Pos**	**2.70**		**3.26**									**3.03**	**Pos**
MS 2^‡^	**Pos**	**2.85**	**2.67**	**/**	**/**			**/**	**/**		**2.56**	**/**	**/**	**Pos**
MS 3	Neg					**3.7**	**3.48**	**6.01**	**3.86**					**Pos**
MS 4^‡^	Neg				**3.15**				**3.32**					**Pos**
MS 5^‡^	Neg					**4.1**	**4.58**	**7.30**	**6.18**					**Pos**
MS 6	Neg				**2.68**									**Pos**
MS 7	Neg								**5.47**					**Pos**
MS 8	Neg								**4.74**					**Pos**
MS 9	Neg													Neg
MS 10	Neg													Neg
MS 11	Neg							**3.77**						Neg
MS 12	Neg										**3.72**			Neg
MS 13	Neg													Neg
MS 14	Neg													Neg
MS 15	Neg													Neg
MS 16	Neg													Neg
MS 17	Neg													Neg
MS 18^‡^	Neg				**3.00**									Neg
MS 19^‡^	Neg		**/**	**/**			**/**	**/**			**/**	**/**		Neg
MS 20^‡^	Neg	**/**	**/**			**/**	**/**	**6.04**		**/**	**/**			Neg
MS 21	Neg													Neg
Median JCPyV load**		***2.77***	***2.67***	***3.26***	***3.00***	***3.90***	***4.03***	***6.03***	***4.74***		***3.14***		***3.03***	
p value^§^									***0.04***					

**pos**: positive; **neg**: negative.

* 78 urine, 78 plasma and 78 PBMCs samples were collected at baseline (t0) and after 4 (t1), 8 (t2), and 12 (t3) months of therapy.

† 2-step virus-like particle-based enzyme-linked immunosorbed assay (ELISA) (STRATIFY JCV®) was performed at t0 and t3, to detect specific anti-JC virus antibodies in serum of the 21 enrolled subjects.

** JCPyV loads values are expressed as log10 genome equivalent (gEq)/mL in urine and in plasma, and as log10 gEq/10^6^ cells in PBMCs and they are indicated in boldface.

§ By χ2 test: positive STRATIFY JCV® patients after 1-year of natalizumab treatment vs negative STRATIFY JCV® patients after 1 year of natalizumab treatment. p value < 0.05 only for JCPyV DNA positive urine samples.

‡ patient MS2 suspended natalizumab for allergic reaction; patients MS19 and MS20 showed poor compliance; patient MS18 suspended the biological therapy for uterine cancer and papillomatosis after 11 months of natalizumab infusions; patient MS4 did not perform 2 natalizumab infusions because of *Escherichia coli* urinary infection; patient MS5 showed clinical relapse during follow up.

Regarding the Cohort2, the demographic and clinical characteristics of the CIRDs patient are shown in Table 2. For this cohort, we collected only the baseline (t0) patients’ features and the JCPyV DNA q-PCR results in urine, plasma and PBMCs samples obtained at t0. About baseline (t0) JCPyV q-PCR detection, we observed JCPyV DNA in 14/22 urine samples with this distribution among the three specific CIRDs considered: 7 in RA, 3 in ankylosing spondylitis (AS) and 4 in psoriatic arthritis (PsA) (Table 3). Plasma and PBMCs samples were all negative for JCPyV DNA detection at t0. No significant differences were found between PsA and RA patients for gender, age, patient and physician global assessment, Health Assessment Questionnaire (HAQ) score, Disease Activity Score with a 28-joint count C-reactive protein-related (DAS28-CRP) and erythrocyte sedimentation rate-related (DAS28-ESR). Moreover, no significant differences were observed concerning the type of concomitant Disease Modifying Anti-Rheumatic Drugs (DMARDs) and disease duration, although disease duration was numerically, but not significantly, higher in RA patients (Table 3).

**Table 2:** Main features of patients with chronic inflammatory rheumatic diseases (CIRDs) (n=22)

**Characteristics**	
Male/Female (n)	7/15
Age (years; median/range)	53/36-79
Diagnosis: PsA/RA/AS	11/8/3
Symptoms onset (months ago; median/range)	75/24-420
CRP (mg/dl; median/25^th^-75^th^ percentile)	0.09/0-1.225
ESR (mm/h; median/25^th^-75^th^ percentile)	18.5/13.25-33.75
HAQ (0-3; mean/SD)	1.275 ± 0.82
DAS28-ESR (mean/SD)*	4.6 ± 1
DAS28-CRP (mean/SD)*	4.05 ± 0.99
Physician’s global assessment of disease activity (0-100 mm, VAS; mean/SD)*	56.3 ± 28.2
Patient’s global assessment of disease activity (0-100 mm, VAS; mean/SD)*	59.6 ± 30.5
BASDAI (1-10; mean/SD)**	7.1 ± 3
Concomitant DMARDs (n/%)	10/45.4
Concomitant DMARDs and glucocorticoids (n/%)	4/18.2
Concomitant glucocorticoids (n/%)	10/45.4
No concomitant treatment (n/%)	6/27.3

**PsA**: psoriatic arthritis; **RA**: rheumatoid arthritis; **AS**: ankylosing spondylitis; **CRP**: C-reactive protein; **ESR**: erythrocyte sedimentation rate; **HAQ**: Health Assessment Questionnaire; **SD**: standard deviation; **DAS28**: 28-joint Disease Activity Score; **VAS**: visual analog scale; **BASDAI**: Bath Ankylosing Spondylitis Diseease Activity Index; **DMARDs**: disease modifying anti-rheumatic drugs.

*For patients with RA and PsA.

**For patients with AS.

**Table 3:** **Baseline (t0) features and *JC viruria *of 21 MS, 18 CIRDs and 22 CD patients**

***Pathology***	***Cohort1 *MS**	***Cohort2***				***Cohort3 *CD**	***Control group***
		**PsA**	**RA**	**AS**	**CIRDs**		
**Female /** Male	**10** / 11*	**5** / 6	**8** / 0	**2** / 1	**15** / 7	**8** / 10	**10** / 9
**JCPyV+** / JCPyV-	**2** / 18*	**4** / 7	**7** /1	**3** / 0	**14** / 8	**6** / 12	**5** / 14
JCPyV load^†^	**3.90 **(3.70 - 4.10)	**7.27 **(4.23-8.09)	**6.92 **(4.15-8.13)	**7.25 **(5.08-7.68)	**7.21 **(4.15 - 8.13)	**6.30 **(4.85-8.85)	**5.75 **(3.54-8.43)
Age^†^	**37** (19–49)	**53** (46–72)	**53.5** (36–79)	**47** (38–54)	**53** (36–79)	**15** (8–22)	**35** (25–47)
Symptoms onset, months ago^†^	**72 **(12–300)	**60 **(24–312)	**108** (52–156)	**120 **(36–420)	**75** (24–420)	*nd*^‡^	
Disease activity^†§^	**2 **(0–4)^§^	**4.11 **(2.46–4.61)^§^	**4.18** (2.50–5,89)^§^	**7,3 **(4–10)^§^		**> 30**^§^	

**MS:** multiple sclerosis**; PsA**: psoriatic arthritis; **RA**: rheumatoid arthritis; **AS**: ankylosing spondylitis; **CD**: Crohn's disease; **CIRDs**: chronic inflammatory rheumatic diseases.

† For each category the median values (range) are indicated. In particular JCPyV loads values are expressed as log10 genome equivalent (gEq)/mL in urine and in plasma, and as log10 gEq/10^6^ cells in PBMCs.

§: Disease Activity is indicated by scale, score or index specific for each disease assessed: for Cohort1 MS, Kurtzke Expanded Disability Status Scale (EDSS) is used. This scale ranges from 0 (normal neurological examination) to 10 (confined to bed); for PsA and RA (Cohort2), Disease Activity Score with a 28-joint count related to C-reactive protein (DAS28-CRP) is used, with a high score indicating more active disease; for AS (Cohort2), Bath Ankylosing Spondylitis Disease Activity Index (BASDAI) is used. This index ranges from 0 (no activity) to 10 (maximum activity); for Cohort3 CD, Pediatric Crohn's Disease Activity Index (PCDAI) is used (score ≤10: inactive disease; 10–30: mild disease; >30: moderate to severe disease).

* At t0, 1 patient (out of the 21 multiple sclerosis patients enrolled) do not perform the urine and blood sampling.

‡ nd: no data.

Regarding the Cohort3, eighteen patients (10 males, 8 females; mean age ± standard deviation (std. dev.): 14.61 ± 4.36 years) with refractory active CD and thus aimed at infliximab treatment, were enrolled. After enrollment, clinical characteristics of CD disease were recorded and clinical activity, measured with the Pediatric CD Activity Index (PCDAI), was > 30 (Table 3). During 16-months follow up, improvements were observed in each patient based on the physician's global evaluation of amelioration compared with baseline (data not shown).

### Baseline (t0) JCPyV DNA detection in plasma and urine samples

**Figure 1:**
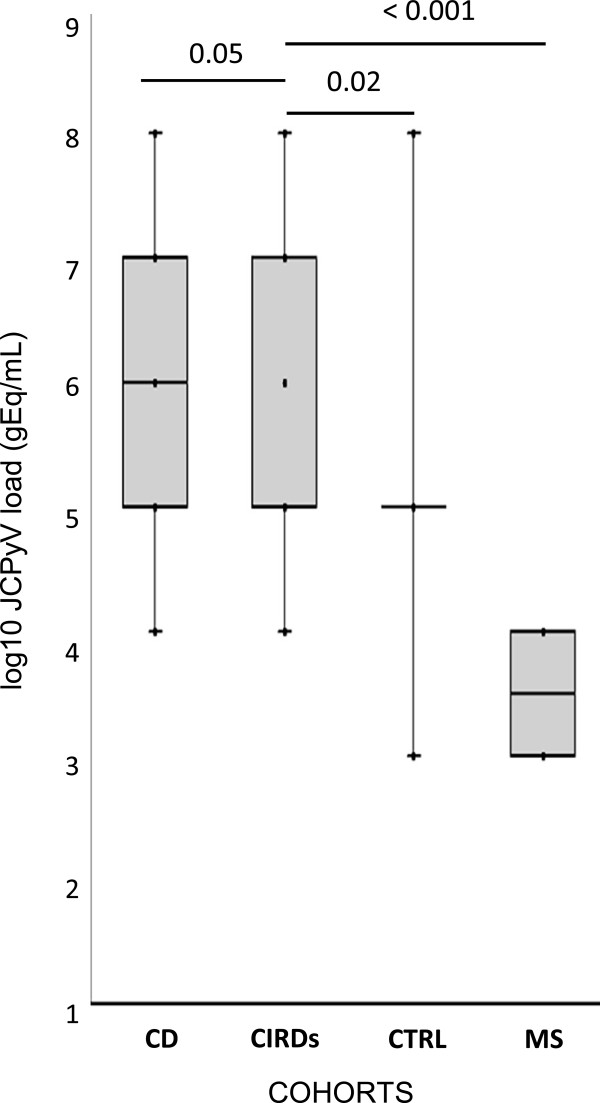
***Comparison of baseline (t0) JC viruria in 21 MS, 18 CIRDs and 22 CD patients.*** JCPyV DNA [(median log10 JCPyV load (range)] was found in 2/21 multiple sclerosis patients [3.90 gEq/mL (3.70-4.10)], in 14/22 CIRDs patients [7.21 gEq/mL (4.15-8.13)] and in 6/18 CD patients [6.30 gEq/mL (4.85-8.85)]. Moreover 5/19 healthy individuals showed JCPyV DNA in urine [5.75 gEq/mL (3.54-8.43)]. Comparing these data of JC viruria, we observed that CIRDs patients presented a JC viruria significantly higher than that presented by patients with MS or with CD and by healthy individuals. JCPyV loads values are expressed as log10 genome equivalent (gEq)/mL. Comparisons were performed using non parametric Mann–Whitney U-test and statistically significant p values (< 0.05) were indicated.

Regarding the baseline (t0) JCPyV DNA detection by q-PCR in plasma samples, collected from the three enrolled Cohorts, we detected JCPyV load [median log10 (range)] in 2/21 MS patients [2.77 gEq/mL (2.70-2.85)], in none CIRDs patients and in 6/18 CD patients [4.93 gEq/mL (3.06-6.58)]. Moreover, only 1/19 healthy individual presented JCPyV DNA in plasma (2.83 gEq/mL). Comparing the JCPyV DNA results obtained in plasma, we did not observe significant difference among the three cohorts, maybe because the number of patients enrolled was too small. On the other hand, regarding the baseline (t0) JCPyV DNA detection in urine samples, collected from the three Cohorts, we found JCPyV load [median log10 (range)] in 2/21 MS patients [3.90 gEq/mL (3.70-4.10)], in 14/22 CIRDs patients[7.21 gEq/mL (4.15-8.13)] and in 6/18 CD patients [6.30 gEq/mL (4.85-8.85)]. Moreover, 5/19 healthy individuals showed JCPyV DNA in urine [5.75 gEq/mL (3.54-8.43)]. Comparing these data of JC viruria, we observed that CIRDs patients showed a JC viruria significantly higher than that observed in patients with MS or with CD (p < 0,05) and in healthy individuals (Figure 1). These data could lead us to conclude that the CIRDs inflammatory scenario could favor the JCPyV shedding in the urine but not the JC viremia appearing. Finally, no patient or healthy individual showed JCPyV DNA detectable in PBMCs samples at t0.

### Anti–JCPyV antibodies in serum and longitudinal assessment of JCPyV DNA in urine, plasma and PBMCs samples collected from MS patients

The anti-JCPyV antibodies ELISA assay (STRATIFY JCV®) was performed only at the study enrollment (t0) and after one year of natalizumab infusions (t3) in serum samples of the 21 enrolled MS patients. At t0 only 2/21 (9%) individuals resulted seropositive for JCPyV. At t3, the number of positive STRATIFY JCV® patients moved from 2 to 8 (Table 1). We found a statistical significant difference between positive and negative STRATIFY JCV® patients regarding the number of months from symptoms onset: positive STRATIFY JCV® patients have a number of months from symptoms onset higher than those of negative STRATIFY JCV® patients (mean months from symptoms onset ± std dev: 114 ± 104.22 vs 87.69 ± 80.26) (p < 0.05) (data not shown). On the other hand, JCPyV DNA was detected by q-PCR in urine, plasma and PBMCs samples collected at baseline (t0) and after 4 (t1), 8 (t2), and 12 (t3) months of natalizumab infusions. From data obtained JCPyV DNA was observed in plasma in 5/21 cases, in urine in 7/21 individuals and in PBMCs in 3/21 cases (Table 1).

**Figure 2:**
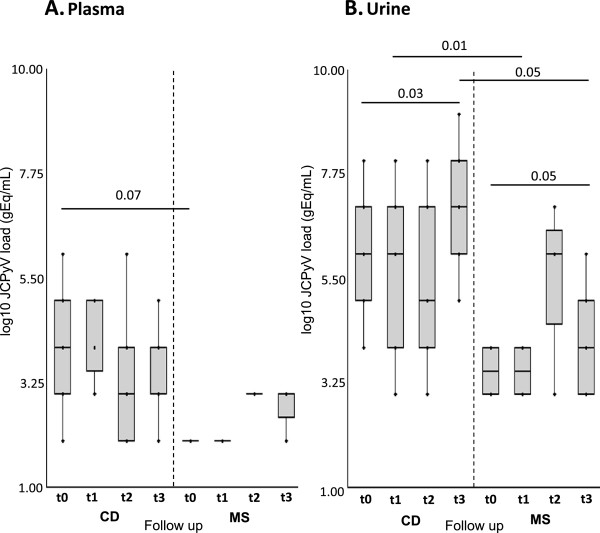
***Comparison of JC viremia and viruria in 1-years follow-up: 21 MS vs 18 CD patients. *(A.)** Comparing JC viremia follow up of MS *vs* CD patients, the viremia of CD patients during the follow-up is numerically higher than that observed in MS patients, although we did not find a statistically significant difference (p = 0.07). **(B.)** Comparing JC viruria follow up of MS patients treated with natalizumab *vs* CD patients treated with infliximab, we observed a persistent viruria both cohorts. However the JC viruria of patients treated with infliximab, was significantly higher than that of patients treated with natalizumab only at t1 (p = 0.01) and at t3 (p = 0.05). Moreover a significant difference between the JC viruria detected at t0 and at t3 in both cohorts was also reported. JCPyV load values are expressed as log10 genome equivalent (gEq)/mL. JCPyV load values of MS cohort and CD cohort are reported in table 1 and table 4, respectively. Comparisons were performed using non parametric Mann–Whitney U-test and p values <0.05 were considered statistically significant. t0: baseline values. t1, t2 and t3: 4, 8 and 12 months of therapy, respectively.

Comparing the results of positive STRATIFY JCV® with JCPyV DNA q-PCR data obtained in plasma, PBMCs and urine collected from MS patients during 1 year of natalizumab treatment, we observed that 2 patients with a positive STRATIFY JCV® at t0, showed both viremia at t0 and then 1 at t1 and one at t2, but no viruria. Moreover, in the same 2 patients, JCPyV DNA was also detected in PBMCs at t3 and t1 (Table 1). Conversely, in the other 6 patients with positive STRATIFY JCV® after one year of treatment with natalizumab, 2 patients showed viruria all along the follow-up but not viremia; 3 patients presented viruria only at t3 and 1 of them showed also viremia at t3; finally, 1 patient showed only viremia at t3. No significant differences were observed in JCPyV viral load in plasma (log10 gEq/ml) when baseline (t0) results (median: 2.77, range: 2.70-2.85) were compared to data obtained at the different time of follow-up (t1: 2.67; t2: 3.26; t3: 2.91, 3.15-2.68) (Table 1 and Figure 2A). Conversely, a significant difference was observed in JCPyV viral load in urine (log10 gEq/ml) when baseline (t0) results (median: 3.90, range: 3.70-4.10) were compared to data obtained at t3 (median: 4.74, range: 3.32-6.18) (p = 0.05) (Table 1 and Figure 2B).

On the other hand, among the 13 patients with persistent negative STRATIFY JCV® after 1 year of natalizumab treatment, 1 patient showed viremia only at t3, 2 patients presented viruria only at t2, and 1 patient showed JCPyV DNA detectable in PBMCs samples at t1. The remaining 9 negative STRATIFY JCV® patients were negative to DNA detection in plasma, PBMCs and urine samples all along the follow-up.

It can also be observed a significant association between JC viruria at t3 and a positive STRATIFY JCV® after 1 year of natalizumab respect to the 13 patients with persistent negative STRATIFY JCV® after 1 year of natalizumab treatment (p = 0.04) (Table 1).

Finally, in this cohort JCPyV load in urine was always significantly higher than that detected in plasma (p < 0.001) and in PBMCs samples (p = 0.02). No difference was observed between the JCPyV loads detected in plasma (median: 2.85, range: 2.67-3.26) and PBMCs samples (median: 3.03, range: 2.56-3.72) respectively (p = 0.83) (data not shown).

STRATIFY JCV® results and longitudinal analysis of JCPyV DNA detection in plasma, urine and PBMCs samples in this cohort were shown in Table 1.

### Longitudinal assessment of JCPyV DNA in urine and plasma samples and intestinal biopsies collected from CD patients

The follow-up of the JCPyV DNA detection in urine and plasma samples and intestinal biopsies collected at baseline (t0) and after 4 (t1), 8 (t2), 12 (t3) and 16 (t4) months of infliximab treatment was performed by q-PCR in Cohort3.

Results on plasma samples showed the presence of JCPyV in 6/18 CD patients at t0. At t1 in Cohort3, the patients with viremia were 7/18, whereas at t2 and t3, we observed a decrease of the JCPyV positive plasma specimens up to 5/18 and this number remained constant at t4 (Table 4). These data showed that the number of patients with JC viremia in Cohorts 3 remain constant during the follow-up (p > 0.05). Comparing the JC viral loads in plasma at each time of follow-up (Table 4), we did not observe a significant difference (p > 0.05), as shown in Figure 2A.

**Table 4:** JCPyV DNA detection in samples collected from 18 CD patients during 1 year of infliximab

**Plasma (log10 gEq/mL)**					
Time*	**t0**	**t1**	**t2**	**t3**	**t4**
**JCPyV+** / JCPyV-	**6** / 12	**7** / 11	**5** / 13	**5** / 13	**5** / 13
median JCPyV load (range)	4.93	5.30	3.08	3.63	4.24
	(3.06-6.58)	(3.78-5.59)	(2.58-6.11)	(2.76-5.35)	(3.92-5.54)
**Urine (log10 gEq/mL)**					
**JCPyV+ / JCPyV-**	**6** / 12	**10** / 8	**6** / 12	**6** / 12	**6** / 12
median JCPyV load (range)	6.30	6.12	5.85	7.47	6.70
	(4.85-8.85)	(3.17-8.37)	(4.30-8.15)	(5.63-9.39)	(3.85-7.85)
**Ileum (log10 gEq/10**^**6 **^**cells)**					
**JCPyV+ / JCPyV-**	**10** / 8			**7** / 11	**4** / 14
median JCPyV load (range)	4.29			5.85	5.07
	(3.54-6.57)			(4.34-8.48)	(3.81-7.15)
**Colon rectum (log10 gEq/10**^**6 **^**cells)**					
**JCPyV+ / JCPyV-**	**8** / 10			**4** / 14	**4** / 14
median JCPyV load (range)	3.66			4.85	4.25
	(3.10-4.67)			(3.52-6.08)	(3.38-4.78)

*We collected 90 plasma and 90 urine samples from CD patients at the following times of sampling: at the time of recruitment (t0) and at 4, 8, 12 and 16 months from t0 (t1, t2, t3, and t4 respectively). 54 ileal and 54 colon–rectal biopsies were recruited at t0, t3 and t4 within the same cohort.

The viral DNA was found in 6/18 urine at t0 with a median JCPyV load (range) of 6.30 gEq/mL (4.85-8.85) and four months later (t1), the number of patients with viruria increased up to 10/18 (median: 6.12 gEq/mL; range: 3.17-8.37), whereas at t2, a reduction of this number was observed (6/18). Finally, we found JCPyV in 6 urine out of 18 CD patients both at t3 and at t4 (Table 4). No significant difference in the number of patients with JC viruria was found during the follow-up (p > 0.05). Comparing the urinary JCPyV loads obtained at each time of follow-up, we observed that viruria significantly increased in the patients treated with infliximab at t3 respect to the value obtained at t0 (p = 0.03). In fact, at t3 the median JCPyV load reached the value of 7.47 log gEq/mL (range: 5.63-9.39) respect to the baseline value of 6.30 gEq/mL (range: 4.85-8.85) (Figure 2B).

Finally, JCPyV load in plasma was always significantly lower than that observed in urine (p < 0.001) (data not shown).

Concerning JCPyV detection in the ileal and colon-rectal biopsies in the cohort of CD, results showed that at t0 JCPyV was found in 10/18 ileal specimens and in 8/18 colon-rectal biopsies. At t3, we found JCPyV in 7/18 ileal biopsies and in 4/18 colon-rectal biopsies. At t4 the number of JCPyV-positive ileal specimens decreased to 4/18, whereas the number of JCPyV-positive colon-rectal biopsies remained constant (Table 4). From these data we observed a numerical decrease in the number of patients with JC viral load in both colon-rectal and ileal biopsies after 16 months of treatment with infliximab, although this decrease was not statistically significant **(**p > 0.05). Moreover, a constant JC viral load value was found in both type of biopsies during the follow-up and the JCPyV load in ileal biopsies was shown to be numerically higher that that observed in colon-rectal biopsies, although the difference was not statistically significant (p = 0.09) (data not shown).

### JC viruria and viremia 1 years follow-up: MS patients treated with natalizumab vs CD patients treated with infliximab

Comparing the JCPyV loads in plasma samples obtained from MS and CD patients during the follow up, we observed that JC viremia in CD patients is numerically higher than that of MS patient, although we did not find a statistically significant difference (p = 0.07), probably because the number of patients enrolled was too small (Figure 2A).

Conversely, comparing the JCPyV loads in urine samples of MS patients treated with natalizumab with that of CD patients treated with infliximab, we observed a persistent viruria in both Cohorts, although the JCPyV viruria of patients treated with infliximab was significantly higher than that of patients treated with natalizumab at t1 (p = 0.01) and at t3 (p = 0.05) (Figure 2B).

### JCPyV VP1 sequence analysis

We performed the JCPyV VP1sequence analysis by automatic sequencing of PCR product corresponding to JCPyV VP1 regions, in order to identify JCPyV genotype featuring our enrolled Cohorts. In all 3 Cohorts we observed a prevalence of genotypes 1A and 1B and it is also important to note that in each patient we never observed a co-infection with different strains of JCPyV. In particular, among the 21 MS patients, we observed JCPyV Type 1A, Type 1B and Type 4 in 10, 9 and 2 individuals, respectively. Moreover, JCPyV Type 1A was also observed in 10/18 CD patients and 9/22 CIRDs patients, whereas the other individuals (8/18 CD patients and 13/22 CIRDs patients) showed a JCPyV genotype 1B (data not shown).

### JCPyV NCCR sequence analysis

**Figure 3:**
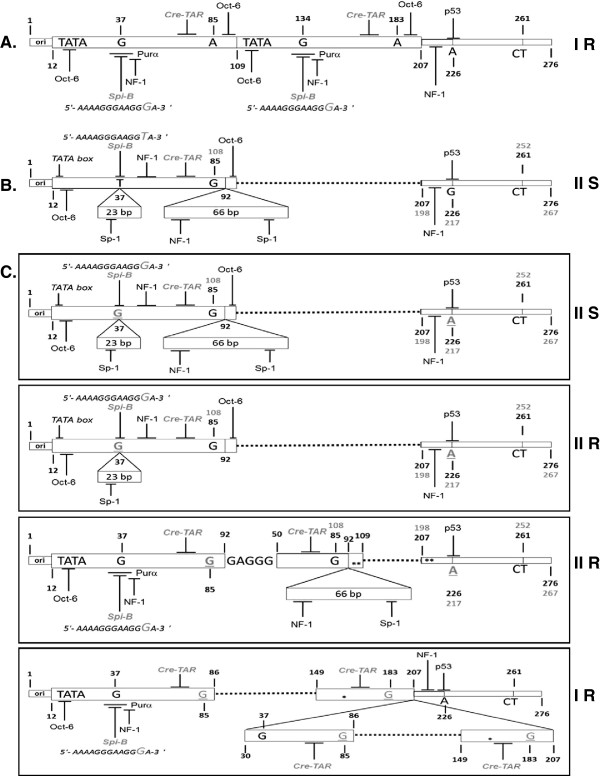
***JCPyV NCCR sequence analysis in 21 MS and 18 CD patients. *(A.)** Type IR has repeats of 98bp unit, also known as sequence composed by boxA (25 bp), boxC (55 bp) and boxE (18 bp), as seen in the JCPyV prototype Mad-1 [35,36], without 23- and/or 66-bp inserts, also known as boxB and boxD respectively. **(B.)** Type IIS has a single 98 bp unit, with one 23- and one 66-bp insert, as seen in the archetype CY [35,38]. The NCCR type IIS is also known as sequence composed by boxes A, B, C, D and E. Each 98bp unit is represented by an open box. The 23 bp and 66 bp inserts are represented as open boxes labeled “23 bp” and “66 bp,” respectively. The nucleotide numbering of Mad-1 NCCR is indicated in black bold font, whereas the nucleotide numbering of CY NCCR is reported in grey bold font. In all NCCR sequences is present a 69 bp sequence, called box F, starting from nucleotide 207. **(C.)** A NCCR Type IIS was found in 8/28 plasma, 7/21 ileal and 5/16 colon-rectal biopsies, collected from CD patients. A NCCR Type IIR with a boxD deletion was found in 4/21 ileal and 1/16 colon-rectal biopsies. Another NCCR Type IIR, composed by a duplication of a 98 bp unit and a 66 bp insert, was found in 2/3 PBMCs of 2 MS patients. Finally, a NCCR Type IR sequence was found in 2/16 colon-rectal biopsies. Dotted lines represent deletions or regions not present. Asterisks represent single nucleotide point mutations or deletions. Black bold letters indicate nucleotides and underlined letters in grey bold font indicate the relative point mutations. The types of NCCR are indicated. The main cellular factor binding sites are also reported. Ori: replication’s origin.

The NCCR forms were classified into four types as previously described [35]. Type I-S has a single 98 base-pair (bp) unit, also known as sequence composed of boxA (25 bp), boxC (55 bp) and boxE (18bp). Type I-R has repeats of this 98 bp unit, with various deletions, as seen in the JCPyV Mad-1 (GenBank n^o^: J02227) and Mad-4 [36,37] strains; both of these types have no 23- and/or 66 bp inserts, also known as boxB and boxD, respectively (Figure 3A). Conversely, type II-S has a single 98 bp unit and one 23- and one 66 bp insert, as seen in the archetype CY [38], also known as sequence composed of boxes A (25 bp), B (23 bp), C (55 bp), D (66 bp) and E (18 bp) (Figure 3B). Finally, type II-R has repeats of this 98 bp unit and inserts with various mutations, also called rearranged NCCRs. Furthermore, in all NCCR sequences a 69 bp sequence is always present, called boxF, starting with the 207 nucleotide considering the nucleotide numbering based on PML-associated variant Mad-1 NCCR sequence [36].

In MS patients, the archetypal variant type IIS has been found in all samples analyzed, with the exception of 2 rearranged NCCR sequences type IIR isolated from PBMCs of the only 2 MS patients, resulted STRATIFY JCV® positive t0 and t3. These rearranged NCCR showed a duplication of a 98bp unit and a 66bp insert, resulting in a boxB deletion with a 37 T to G nucleotide transversion (37 T→G) into the Spi-B binding site, followed by a duplication of the boxC and boxD insertion (Figure 3C and Table 5B).

**Table 5:** NCCR analysis in samples collected from Crohn’s Disease and Multiple Sclerosis patients

**A. Crohn’s Disease patients**					
**Samples**	**N° JCPyV positive samples**	N° archetype* (%) (IIS)^**†**^	37 T→G and 217 G→A** (IIS)^**†**^	**N° rearranged NCCR sequences (%)**	
				37 T→G , 217 G→A** and boxD deletion (IIR)^**†**^	***Mad1-like sequence (IR)***^**†**^
Urine	34	34 (100%)	0	0	0
Plasma	28	20 (71%)	8 (29%)	0	0
Ileum	21	10 (47%)	7 (33%)	4 (20%)	0
Colon-rectum	16	8 (50%)	5 (32%)	1 (6%)	2 (12%)
Total (%)	99	72 (72%)	20 (21%)	5 (5%)	2 (2%)
**B. Multiple Sclerosis patients**					
**Samples**	**N° JCPyV positive samples**	N° archetype* (%) (IIS)^**†**^	**N° rearranged NCCR sequences (%)**		
			***duplication of a 98bp unit and 66bp insert (boxD)(IIR)***^**†**^		
Urine	13	13 (100%)	0		
Plasma	7	7 (100%)	0		
PBMC	3	1 (33%)	2 (67%)		
Total (%)	23	21 (91%)	2 (9%)		

* JCPyV archetype CY [38] with random occurrence of few point mutations and constant G to A transition at position nucleotide number 217 in the boxF.

** T to G transversion at position nucleotide number 37 in the box A within Spi-B binding site, and G to A transition at position nucleotide number 217 in the boxF considering the nucleotide numbering based on non-pathogenic JCPyV archetype CY [38].

† Jensen and Major (2001) NCCR forms classification is also reported [35].

Moreover, all sequences found in the 14 urine samples with JC viruria and collected at t0 from CIRDs patients, showed archetype structure type IIS (Figure 3B), with only a 217 G to A nucleotide transition (217 G→A) in the boxF.

Regarding the sequences analysis of NCCR found in plasma, urine and intestinal biopsies of patients with CD treated with infliximab, the 72% of all sequences analyzed showed an archetypal structure type IIS. A NCCR Type IIS with a 37 T→G within Spi-B binding site, and a 217 G→A in the boxF, was found in 8/28 (29%) plasma, 7/21(33%) ileal and 5/16 (32%) colon-rectal biopsies. A rearranged JCPyV NCCR Type IIR with the two point mutation previously described and a boxD deletion was found in 4/21 (20%) ileal and 1/16 (6%) colon-rectal biopsies. Finally, a rearranged JCPyV NCCR Mad-1-like type IR sequence, composed by repeats of truncated 98bp unit, resulting in deletion of the boxB followed by a tandem repeat sequence constituted by a partial boxC duplication, a boxD deletion and an integral boxE, was found in 2/16 (12%) colon-rectal biopsies. All these rearranged sequences were found only in the intestinal biopsies and plasma samples collected at t3 and t4 from seven pediatric CD patients treated with infliximab. All these seven patients presented a value of PCDAI> 40 at t0. The rearranged JCPyV NCCR Mad-1-like type IR sequence was instead found at t4 (after 16 months of infliximab treatment) in 2 colon rectal biopsies collected from 2/7 patients indicated above (Figure 3C and Table 5A).

## Discussion

In the last decades, PML onset during treatment with biological agents has made imperative to assess the risk of PML associated with these therapies, in order to achieve a well-informed risk/benefit decision-making. However, the epidemiology of PML in such settings remained difficult to define, because PML is rare and likely underdiagnosed and because the nature of the risk associated with biological therapy is widely debated [29]. Firstly, it has been important to understand whether the specific inflammatory scenario of different immune-mediated diseases could affect the JC virus reactivation from its sites of latency. Several immune-mediated diseases are characterized by common alterations of the cell T helper (Th) 1 versus Th2 and interleukin (IL) -12/TNF versus IL-10 balance. In particular, in RA, MS and CD, this balance is skewed toward Th1 with an excess of IL-12 and TNF production, whereas the SLE is generally associated with a Th2 shift and an excessive production of IL-10 [39]. In fact, it was over-documented that SLE patients appear to have a particular susceptibility to the development of PML [26-29]. In addition, MS patients with natalizumab-associated PML were distinguished from all other subjects because they had JCPyV-specific CD4+ T cell responses uniquely dominated by IL-10 production. Thus, changes in T cell-mediated control of JCPyV replication may be a risk factor for developing PML [13]. Moreover, in MS and CIRDs patients, glucocorticoids treatment is associated with increased plasma IL-10 secretion [39]. These observations suggested that virus is kept at bay by cell-mediated immunity in healthy subjects, although JCPyV-specific antibodies are present in about 58% of healthy individuals [40-42]. On the basis of our results, comparing the JCPyV DNA detection in plasma at the baseline, we did not observe significant differences among the three Cohorts, probably because the number of patients enrolled was too small. Conversely, comparing baseline JCPyV DNA detection in urine samples, we observed that CIRDs patients showed a JC viruria significantly higher than that observed in patients with MS or with CD (p<0.05) and in healthy individuals. These data could lead us to conclude that the CIRDs inflammatory scenario could favor the JCPyV shedding in the urine but not the JC viremia appearing. Taking into account the results obtained and the assumptions previously made, we also observed that 7 out of 14 CIRDs patients, who shed JCPyV in the urine, underwent concomitant therapy with glucocorticoids, whereas no MS patients were treated with glucocorticoids before natalizumab treatment. Moreover, it was also well known that the standard treatment of CD patients includes the use of budesonide and prednisone. Therefore, it is reasonable to assume that the use of glucocorticoids, as concomitant therapy in the treatment of immune-mediated diseases, could have favored the JCPyV reactivation in the kidney. In fact, as previously mentioned, treatment with glucocorticoids is associated with increased plasma IL-10 secretion and thus with an imbalance of cell mediated immunity, that finally may have determined the JCPyV shedding in urine, especially in CIRDs patients. However, this assumption requires a further systematic investigation on correlation between use of glucocorticoids and prevalence of JC viremia and viruria in patients affected by immune-mediated diseases.

To date, there are no methods that can reliably predict which patients have a higher risk of developing PML and no clear-cut associations can be established between JC viremia and viruria and PML. In the attempt to bypass this problem, a new assay (STRATIFY JCV®) was suggested to be a sensitive tool to identify JCPyV seroconverted patients [23], especially among MS patients treated with natalizumab. Therefore, regarding the monitoring of the asymptomatic reactivation of JCPyV in peripheral blood and urine of MS patients treated with natalizumab for 1 year, some authors [43,44] reported the frequent detection of JCPyV reactivation in the urinary tract after therapy. In agreement with these authors, in this study a significant difference was observed in JCPyV viral load in urine when baseline (t0) results were compared to data obtained at t3 (p = 0.05). It can also be observed a significant association between JC viruria at t3 and a positive STRATIFY JCV® after 1 year of natalizumab respect to the 13 patients with persistent negative STRATIFY JCV® (p = 0.04). As other Authors have suggested [24], complementary tests of JCPyV DNA in urine seems to be useful to stratify the PML risk, since the detection of urinary viral DNA identifies JCPyV infected subject when antibodies are still undetectable. This phenomenon may depend on the inter-individual difference mounting a humoral immune response against JC virus or on the 2.5% of false negative estimated for the STRATIFY JCV® test [23]. However, to date these observations were not replicated in other analyses [25]. Conversely, JCPyV DNA in plasma or PBMCs may not be useful in PML risk stratification. It was debated whether the detection of JCPyV in blood results in a higher likelihood to develop PML, but it was often observed that JCPyV DNA can be detected in blood in healthy individuals and that the JCPyV load in blood is usually very low even in patients with PML diagnosis [45]. Moreover, no PML cases have ever been reported in JCPyV seronegative individuals [14], and the estimated PML incidence in JCPyV seronegative patients is extremely low (0.09 cases per 1000 patients) [12]. Thus, humoral immunity alone seems to be not able to control JCPyV replication [46] as well as cellular immunity, since JCPyV shedding in urine of immunocompetent individuals is very common [40]. Although our results were obtained in a small cohort of patients and need to be replicated and expanded, they suggest that JCPyV replication with shedding in urine could be not necessarily a sign of T cell dysfunction, but instead reflects differences in anatomic location and/or accessibility to T cells, as other authors also suggested [25].

Finally, in CD patients we observed that the number of patients with JC viremia and/or viruria remains constant during the follow-up, but comparing the urinary JCPyV loads obtained at every time of follow-up, we observed that viruria significantly increased in patients treated with infliximab at t3 respect to the value obtained at t0 (p=0.03). This result puts in highlight a higher viral replication in the renal tubular epithelial cells with a shedding of the virus in the urine after 1 year of infliximab treatment. Moreover, a constant JC viral load value was found in intestinal biopsies during the follow-up. Therefore, although infliximab was not implicated in JCPyV reactivation directly, it seems to interfere with the fine balance between the anti-inflammatory activity of this biologic and the host immune surveillance [33]. Since the use of infliximab in CD patients blocks the TNF and interferes with the recruitment of lymphocytes causing a decreasing of interferon (IFN)-γ levels involved in the anti-viral state control, JCPyV could leave its latent state within the enteric glial cells [47] and could infect the intestinal epithelium. Furthermore in patients affected by immune-mediated diseases and treated whit biologics, JCPyV reactivation has been observed to be associated with increased plasmatic levels of monokine induced by IFN-γ (MIG), which represents a T cell-attracting chemokine released by eosinophils [48,49]. Conversely, JCPyV could also restart its lytic cycle in other cell types, including the CD34+ haematopoietic precursors, the B cells present in BM and in circulation [50] and the renal tubular epithelial cells [51,52]. This hypothesis could also be supported by the fact that the median viremia reached the value of 5.30 log gEq/mL in the patients treated with infliximab at t1, when the drug achieved its highest effectiveness. Finally, regarding all intestinal biopsies examined, we can hypothesize that JCPyV may undergo reactivation in the gut with a spreading of virions in the bloodstream, according to Selgrad and collegues who speculated that this neurotropic virus may switch its latent state in the enteric neurons on an active lytic infection [47].

The interfering of infliximab in the regulation of the fine balance between anti-inflammatory drug benefit and immune surveillance, was also evidenced by the rearranged NCCR sequences found at t3 and t4 in plasma samples and intestinal biopsies of 7 infliximab-treated CD patients, who showed a PCDAI value >40 at t0. The main part of these sequences showed a CY NCCR structure (Type IIS) with two nucleotide changes, the 37 G→A in the Spi-B binding site and the 217 G→A in the boxF, whereas other 5 sequences showed a JCPyV NCCR structure Type IIR characterized by the two nucleotide changes mentioned above, and a 66bp insert deletion. Moreover, a rearranged JCPyV NCCR sequence Type IR, composed by repeats of truncated 98bp unit, was found at t4 (after 16 months of infliximab treatment) in 2 colon rectal biopsies collected from 2 of the 7 patients indicated above. Conversely, the rearrangement found in PBMC samples of MS patients, treated with natalizumab and resulted STRATIFY JCV® positive t0 and t3, underlines the role of these cells into delivering JCPyV rearranged forms until the brain throughout the blood stream. In fact, these rearranged NCCR (type IIR) showed a duplication of a 98 bp unit and a 66 bp insert, resulting in a boxB deletion with a 37 T→G nucleotide change into the Spi-B binding site. All these rearranged form could result neuro-invasive and they could increase the risk of PML onset in patients treated with biologics: in fact, we found the rearranged JCPyV NCCR sequences after 16 months of infliximab and 1 year of natalizumab, respectively. This hypothesis could be supported by the fact that natalizumab could mobilize CD34+ hematopoietic precursors and B cells, harboring JCPyV in a latent state, and that Spi-B expression in these cells could enhance the JCPyV NCCR rearrangement. In particular we observed that the 37 T→G nucleotide change shifted the typical CY archetype Spi-B binding site (5′-AAAAGGGAAGG**T**A-3′) to the characteristic one of the PML-associated variant Mad-1 (5′-AAAAGGGAAGG**G**A-3′). The finding of this nucleotide change could enhance JCPyV replication because it has been demonstrated that, the Spi-B-binding sites that actively bind the Spi-B protein expressed in JCPyV susceptible cell types, are present in Mad-1 NCCR sequences, but not in the non-pathogenic archetype CY [53]. Additionally, Spi-B have been shown to be up-regulated in glial cells, B cells and hematopoietic progenitor cells in which JCPyV can replicate [54]. Spi-B binding sites in the promoter/enhancer of JCPyV variants are located directly adjacent to TATA boxes that are essential for the transcription of early and late viral genes. Recruitment of the transcription complex TFIID to JC viral promoters by Spi-B is an attractive model for the activation of JCPyV gene expression in the absence of TAg protein [55,56]. Furthermore, the shift of the typical CY archetype Spi-B binding site in the characteristic one of the PML-associated variant Mad-1, in association with the boxD deletion in 5 intestinal biopsies, let us to conclude that JCPyV is attempting to enhance its virulence especially in those cells, such as intestinal cells, that are not permissive to JCPyV replication. These results were more emphasized by the JCPyV NCCR Mad-1-like type IR sequence found in two colon-rectal biopsies, in which an attempt to recombination could have taken place during virus replication. This last rearrangement could be also favored both by the unbalanced immunological state of these patients and by the 16 months of infliximab treatment. However, the 78% of sequences isolated from the three Cohorts enrolled, including all sequences collected from urine samples, showed a CY archetype NCCR structure (Type IIS), with a 217 G→A in the boxF. This point mutation is completely in agreement with other literature data: in fact, this nucleotide change seems to be a common feature of the European strains as just described by Agostini and colleagues [6].

Moreover, we also observed a prevalence of genotypes 1A and 1B among all patients enrolled, without a JCPyV strains co-infection in the same patient. In particular, in the 2 MS patients with a rearranged NCCR sequence type IIR in the PBMCs, we found the JCPyV Type 1A and the Type 4 respectively, whereas the JCPyV Type 1B was observed in the two patients with the rearranged NCCR sequence type IR in colon rectal biopsies. Although other author found a significant association between the JCPyV Type 2 and infliximab treatment in a pediatric CD cohort [57], we did not observed a correlation between rearranged JCPyV NCCR sequences and a particular JCPyV genotype in each enrolled cohort. Conversely, all genotypes isolated were in agreement with the most common genotypes detected in the European population [6,58].

However, future analyses of alterations and acquisitions of unique transcription factor-binding sites will probably offer more insight into the role of these factors in viral pathogenesis. Moreover, B cells have been shown to support the low levels of JCPyV infection and are likely carriers of infectious virus to the brain during reactivation and dissemination, leading to the development of PML [32]. Thus, the further study of activation of Spi-B gene expression in transitional B cells that contain latent virus may be important to understand the real role of viral reactivation in PML pathogenesis.

## Conclusions

The mechanism underlying JC viral replication are nevertheless still unknown, and the influence of natalizumab on JCPyV control is unclear. However, from these preliminary data we can conclude that it is important to understand whether the specific inflammatory scenario in different immune-mediated diseases could affect the JC virus reactivation from its sites of latency. In particular, an imbalance of cell-mediated immunity may have led to a JCPyV urinary shedding significantly higher in patients with CIRDs at t0 respect to MS and CD patients. Moreover, for a more accurate PML risk stratification in patients treated with natalizumab, testing JCPyV viruria seems to be useful to identify patients who harbor JCPyV but with an undetectable JCPyV-specific humoral immune response. Moreover, since the number of CD patients treated with infliximab showed a JC viruria higher than that observed in patients with MS during the follow-up, we could suppose that infliximab encourages JCPyV reactivation respect to natalizumab.

For a better risk stratification in patients treated with biologics, it may also be important to study the JCPyV NCCR genomic regions. In particular, the rearrangement found in the PBMCs of MS patients treated with natalizumab underlying the role of these cells into delivering JCPyV rearranged forms until the brain throughout the blood stream. These rearranged form could result neuro-invasive and they could increase the risk of PML onset in patients treated with natalizumab. This is supported by the fact that natalizumab could mobilize CD34+ hematopoietic precursors and B cells, harboring JCPyV in a latent state, and that Spi-B expression in these cells could enhance the JCPyV NCCR rearrangement. It’s important to understand the molecular mechanisms involved in JCPyV latency and reactivation in peripheral blood cells, because they represents the main pieces in the puzzle of PML pathogenesis.

Moreover, infliximab could also be implicated into the mobilization of CD34+ hematopoietic precursors and B cells, harboring JCPyV in a latent state, although an evident risk of PML onset after the use of this drug was not observed [50]. Conversely, the issue of JCPyV NCCR rearrangement in non-permissive cells, such as intestinal cells in patients with CD treated with infliximab, and its association with cancer is emerging [1]. This obsarvation could be a great starting point to explore the possible JC virus role in in the pathogenesis of colon cancer, considering also the nature of Polyomaviruses, the oncogenic viruses *par excellence*. Finally, we can highlight that our rearranged NCCR sequences could be considered a marker of the JCPyV virulence during the mAb treatment, although none of our examined patients developed PML, and further studies on larger cohorts should be performed. In conclusion, since the number of patients affected by autoimmune disease and treated with biological agents, continues to rise [59], we could take into account that the follow-up of JCPyV DNA detection and the subsequent JCPyV NCCR sequences analysis may be important in order to reduce the risk of PML onset. Further investigation into the molecular interactions that occurs between Spi-B, protein cofactors, and the JCPyV NCCR in cells that support latent infection will offer additional insight into molecular pathogenesis, reactivation from latency in lymphocytes and PML development. Finally, in individuals treated with immunomodulatory drugs, an impairment of the immune surveillance could be an important risk factor for the JCPyV reactivation and the PML onset. Therefore, it is important to focus all our efforts to find the cellular pathways, finely regulated by the host immune system, that lead to the reactivation of the virus in conditions of immunosuppression, since it is virtually impossible to control the JCPyV infection.

## Methods

### Enrolled patients features and study schedule

In this perspective observational study, three dynamic cohorts of autoimmune diseases patients were enrolled:

1. Twenty-one outpatients with a diagnosis of MS (11 males, 10 females) (Cohort1) were enrolled at the Department of Neurology and Psychiatry (Sapienza University of Rome, Italy) between March 2012 to April 2013. Patient’s mean age (± standard deviation) was 35.05 ± 8.12 years; mean disease duration was 98 ± 88 months with a mean Expanded Disability Status Scale (EDSS) at enrollment of 2.05 ± 1.07. Among the 21 patients, 13 (62%) previously underwent immunomodulating therapies (interferon, glatiramer acetate); 1 patient (5%) was treated with immunosuppressive drugs (mitoxantrone) and the other 7 (33%) individuals were not previously treated at all. No significant differences were observed concerning the type of previous therapy and disease duration. All patients fulfilled the Italian Agency of Drug criteria for natalizumab treatment, i.e. they either were affected by a particularly severe disease course in the year prior to therapy (marked clinical worsening, high relapse rate, rapid disability accumulation), or they showed a lack of response to previous immunosuppressive or immunomodulatory therapies. The therapeutic program of the patients of this cohort consisted of 300 mg intravenous (IV), infused over approximately 1 hour, every 4 weeks (1 infusion a month). 78 urine, 78 plasma and 78 PBMCs samples were collected at baseline (t0) and after 4 (t1), 8 (t2), and 12 (t3) months of therapy. Only for Cohort1, after the activation of a centralized service supported by Biogen Idec (STRATIFY JCV®), 2-step virus-like particle-based enzyme-linked immunosorbed assay (ELISA) was performed at t0 and t3, to detect specific anti-JC virus antibodies [23] in serum of the 21 enrolled subjects treated for a 12-month period.

2. Twenty-two outpatients affected by CIRDs (7 males, 15 females) (Cohort2), referred to the Department of Internal Medicine and Medical Disciplines, Rheumatology Unit (Sapienza University of Rome, Italy), were enrolled from January 2013 to April 2013. After obtaining informed consent, basic demographic data including gender, age, diagnosis, date of diagnosis, concomitant medications and disease activity measures were collected at a single time point, just before the initiation of biologic therapy and recorded on a standardized form (Table 2). Patient’s mean age (± standard deviation) was 54.68 ± 10.79 years and mean disease duration was 112.82 ± 97.44 months. Patients with RA, PsA, and AS, classified according to standard criteria [60-62] and designated to start biologic treatment, were consecutively enrolled. Each patient was evaluated by the same rheumatologist. For RA and PsA clinical evaluation included: swollen (SJC) and tender joint count (TJC), patient and physician global assessment on a visual analogue scale (VAS, 0-100 mm), and HAQ [63]. The HAQ scores range from 0-3, with a higher score indicating a higher level of disability [64]. A blood drawing was also performed to evaluate ESR (mm/h) and CRP (mg/l). Disease activity was assessed by the DAS28-CRP and/or the DAS28-ESR, with a high score indicating more active disease [63]. Current disease activity in patients with AS was measured by the Bath Ankylosing Spondylitis Disease Activity Index (BASDAI), ranging from 0 (no activity) to 10 (maximum activity) [65]. 22 urine, 22 plasma and 22 PBMCs samples collected only at baseline (t0) were utilized to compare the baseline JCPyV DNA quantitative detection in each samples type of these individuals with those of MS and CD patients.

3. Eighteen patients (10 males, 8 females; mean age ± standard deviation: 14.61 ± 4.36 years) (Cohort3) with refractory active CD and thus aimed at infliximab treatment, referred to the Pediatric Gastroenterology and Liver Unit (Sapienza University of Rome, Italy) from January 2010 to December 2012, were enrolled. Inclusion criteria were: CD diagnosis confirmed with usual clinical, endoscopic, radiologic, and/or histologic criteria for at least 6 months; and active luminal or fistulizing disease despite adequate standard treatments. Exclusion criteria were contraindications to infliximab treatment, such as a history of tuberculosis or positive skin test/chest radiogram in the absence of adequate antibiotic prophylaxis, congestive heart failure, demyelinating syndrome, sepsis or abscesses, symptomatic bowel stenoses, history of previous or present cancer, or failure to give informed consent. After enrollment, clinical characteristics of CD disease were recorded, clinical activity was measured with the Pediatric CD Activity Index (PCDAI) [66], collected retrospectively during the visit immediately before infusions according to the Frenz et al. protocol [67], and fistula were assessed according to the Present et al. [68] assessment system. The therapeutic program of the patients of this cohort consisted of three consecutive infusions of infliximab (5 mg/kg) at 0, 2, and 6 weeks for the induction phase. The first infusion was given within 7 days of enrollment into the study. Infliximab was administered intravenously during a period of 2 h, and patients were monitored for heart frequency and for signs of adverse reactions. From each patient, blood samples were taken before infusion, after 8 weeks the beginning of therapy and every 8 weeks thereafter, to determine the activity and nutritional indexes as well as serum variables of renal and hepatic function. All patients were offered a maintenance therapeutic program consisting of repeated infusions of infliximab every 8 weeks for 16 months. The first re-treatment infusion was administrated 8 weeks after the last baseline infusion. Clinical effectiveness was evaluated at week 6: remission was defined by a PCDAI score of less than 10 (for luminal disease) or if all fistulae openings present at baseline were closed (for fistulizing disease). Improvements were determined based on the physician's global evaluation of amelioration compared with baseline [69]. We collected 90 plasma and 90 urine samples from these patients at the following times: recruitment (t0) and at 4, 8, 12 and 16 months from t0 (t1, t2, t3, and t4, respectively). PBMCs samples were not collected in this Cohort because of the small amount of available whole blood. Moreover, 54 ileal and 54 colon–rectal biopsies were collected at t0, t3 and t4.

4. Finally, we enrolled 19 healthy individuals (9 male, 10 females; mean age ± standard deviation: 35.94 ± 6.56 years) (Control Cohort) as control group. For the Control Cohort, 19 urine, 19 plasma and 19 PBMCs samples were obtained from healthy individuals, only at the moment of enrollment (t0).

### Clinical specimens processing and JCPyV DNA extraction

This study was carried out on a total of 186 plasma, 186 urine and 96 PBMCs samples and 108 intestinal biopsies collected at different times from the three cohort as mentioned above. DNA for molecular analysis was extracted from 500 μl of each urine sample, collected without preservatives, using the DNeasy® Blood & Tissue Kit (QIAGEN, S.p.A, Italy) according to the manufacturer’s instructions and stored at -20°C until use. Blood samples, collected in 4-mL Vacuntainer® tubes containing EDTA (BD Becton Dickinson S.p.A, Italia), were centrifuged at 1,376 g/sec for 10 minutes and DNA was extracted from 200 μL of plasma using the DNeasy® Blood & Tissue Kit (QIAGEN, S.p.A, Milan, Italy) and stored at -20°C until use. Peripheral blood mononuclear cells (PBMCs) were isolated from whole blood using the standard Ficoll Hypaque density gradient centrifugation technique [70]; the number of viable leukocytes was determined by trypan blue exclusion. PBMCs DNA extraction was performed on 10^6^ cells by the QIAmp® DNA Blood Kit (QIAGEN S.p.A, Milan, Italy) according to the manufacturer’s instructions and DNA was stored at -20°C until use. DNA yield of all samples was determined by measuring its concentration in the eluate by absorbance at 260 nm and then 0.1-1 μg of total DNA was directly used for PCR amplification assays.

### JCPyV T-Ag real-time PCR (Q-PCR)

Extracted DNA of each samples was analyzed using Q-PCR for the detection and quantification of the JCPyV genome using a 7300 Real-Time PCR System (Applied Biosystems, USA), following a published protocol [71]. Of DNA, 500 ng was used as a template in each reaction, and a 54-bp amplicon in the JCPyV T antigen region was detected. Each sample was analyzed in duplicate, and the viral load results were given as the mean of the two positive reactions. Each run contained a negative control composed of the reaction mixture without DNA template. A positive control consisted of serial dilutions (range, 10^5^ gEq/ml–10^2^ gEq/ml) of a plasmid containing the entire JCPyV genome, on the results of which a standard curve was performed. For urine and plasma specimens, the JCPyV DNA load was expressed as genome equivalents (gEq)/ml of sample and as genome equivalents (gEq)/10^6^ cells of sample for the biopsies. To correct for the variable amount of DNA in different tissue samples, each sample was subjected to simultaneous TaqMan PCR for the housekeeping gene Glyceraldehyde-3-phosphate-dehydrogenase (GAPDH, Accession no. J04038), targeting the region between exons 6 and 8. Results were considered acceptable only in the presence of GAPDH positivity [72].

### JCPyV NCCR PCR

DNA yield was determined by measuring its concentration in the eluate by absorbance at 260 nm, and then, 0.1 to 1 μg of total DNA was directly used in PCR amplification. β-globin PCR was performed on extracted DNA to assess the efficacy of nucleid acid extraction [73]. General precautions, conditions for PCR analysis, and nested-PCR procedures were performed as published [74]. β-Globin-positive samples were amplified in a GeneAmp® PCR System 9700 (Perkin-Elmer Cetus, Emeryville, CA), and all assays included positive (purified recombinant plasmid DNA) and negative (all the PCR components except the template) controls to exclude false-positive and false-negative results [75]. Nested-PCR employed two pairs of primers that anneal to the invariant regions flanking the NCCR of JCPyV [74]. Primers BKTT1 (5′-AAG GTC CAT GAG CTC CAT GGA TTC TTC C-3′) and BKTT2 (5′-CTA GGT CCC CCA AAA GTG CTA GAG CAG C-3′) amplified a 724-bp DNA fragment in JCPyV (Mad-1) [76]. The second pair, JC1 (5′-CCT CCA CGC CCT TAC TAC TTC TGA G-3′) and JC2 (5′-AGC CTG GTG ACA AGC CAA AAC AGC TCT-3′), amplified a portion of the first round PCR product, generating a fragment of 308 bp [77]. Two nanograms of recombinant pGem-1 plasmid DNA containing the complete JCPyV genome, cloned as EcoR1 fragments, were used as positive controls. The PCR products were analyzed on 2% agarose gels by ethidium bromide staining.

### JCPyV VP1 PCR

For JCPyV genotyping a 215 bp fragment was amplified from the VP1 major capsid protein gene using primers JLP-15 (5′-ACA GTG TGG CCA GAA TTC ACT ACC-3′) and JLP-16 (5′-TAA AGC CTC CCC CCC AAC AGA AA-3′) [6]. After initial denaturation at 95°C for 9 min, followed by 40 cycles at 95°C for 40 s, annealing at 63°C for 40 s and extension at 72°C for 40 s, the amplification protocol was concluded with a final extension at 72°C for 7 min. PCR products were analyzed using 2% agarose gel electrophoresis and visualized using ethidium bromide staining [54].

### Sequencing of JCPyV NCCR and VP1 regions

The PCR product corresponding to JCPyV NCCR and VP1 regions were purified prior to sequencing to remove the excess of primers with QIAquick PCR purification kit, according to QIAGEN protocol [74]. DNA sequencing was performed by automatic DNA sequencer (Applied Biosystem, model 370 A), according to the manufacturer′s specifications (Amplicycle Kit, Applied Biosystem). Sequences were organized and analyzed using the Genetic Computer Group Sequence Analysis software package. In particular, all sequences obtained from the amplification of the NCCR region were compared to the JCPyV NCCR sequence of prototype Mad-1 (GenBank: J02227) [36] and of archetype CY [38], whereas those obtained from the amplification of the VP1 region were classified into known genotype/ssubtype analysing the single nucleotide polymorphisms (SNPs) within the amplified VP1 region. To detect the SNPs, by which we have classified our isolates, we aligned our isolated with those reported by Jobes and colleagues in 2001 [78].Sequence alignments were performed with ClustalW2 at the EMBL-EBI website using default parameters [79].

### Data analysis

Data were summarized as medians and ranges or as mean ± standard deviation, as appropriate. If Z test indicated a non-normal distribution, we used non-parametric tests such as Mann–Whitney U test and Kruskal–Wallis test. Categorical data were analyzed by using χ^2^ test and Student's t test. P values <0.05 were considered statistically significant.

## Abbreviations

MS: Multiple sclerosis; CIRDs: Chronic inflammatory rheumatic diseases; CD: Crohn’s disease; PML: Progressive multifocal leukoencephalopathy; JCPyV: Polyomavirus JC; TAg: Large T antigen; tAg: Small t antigen; VP: Viral capsid protein; NCCR: Non-coding control region; mAbs: Monoclonal antibodies; VLA-4: Very late antigen-4; TNF-α: Tumor necrosis factor alpha; RA: Rheumatoid arthritis; VCAM-1: Vascular cell adhesion molecule 1; BM: Bone marrow; FDA AERS: Food and drug administration adverse event reporting system; SLE: Systemic lupus erythematosus; PBMC: Peripheral blood mononuclear cells; Cohort1: Cohort of MS patients; Cohort2: Cohort of CIRDs patients; Cohort3: Cohort of CD patients; q-PCR: Quantitative real time PCR; MIG: Monokine induced by IFN-γ; EDSS: Kurtzke expanded disability status scale; ELISA: Enzyme-linked immunosorbent assay; AS: Ankylosing spondylitis; PsA: Psoriatic arthritis; HAQ: Health assessment questionnaire; ESR: Erythrocyte sedimentation rate; CRP: C-reactive protein; DAS28: Disease activity score with a 28-joint count; DMARDs: Disease modifying anti-rheumatic drugs; std dev: Standard deviation; PCDAI: Pediatric CD activity index; gEq: Genome equivalents; bp: Base pair; 37 T→G: 37 T to G nucleotide transversion; 217 G→A: 217 G to A nucleotide transition; Th: Cell T helper; IL: Interleukin; INF: Interferon; GAPDH: Glyceraldehyde-3-phosphate-dehydrogenase; SJC: Swollen joint count; TJC: Tender joint count; VAS: Visual analogue scale; BASDAI: Bath ankylosing spondylitis disease activity index.

## Competing interests

The authors declare that they have no competing interests.

## Authors’ contributions

VP, AB, LN, ATP designed the experiments; RS, GV, MM, SP, AF, FF, GDN, SC^5^ recruited patients and collected clinical data; AB, EA, DMR, SC^1^, performed experiments and data collection; AB, EA, DMR, SC^1^, SC^7^ were involved in data analysis; VP, AB, RS, LN, GV, ATP interpretation of the data and drafting the manuscripts. All the authors revised and approved the final manuscripts.
